# Prediction of Non-canonical Routes for SARS-CoV-2 Infection in Human Placenta Cells

**DOI:** 10.3389/fmolb.2021.614728

**Published:** 2021-11-08

**Authors:** Flávia Bessi Constantino, Sarah Santiloni Cury, Celia Regina Nogueira, Robson Francisco Carvalho, Luis Antonio Justulin

**Affiliations:** ^1^ Department of Structural and Functional Biology, Institute of Biosciences, São Paulo State University (UNESP), Botucatu, Brazil; ^2^ Department of Internal Clinic, Botucatu Medicine School, São Paulo State University (UNESP), Botucatu, Brazil

**Keywords:** COVID-19, SARS-CoV-2, placenta, virus entry mediator, DPP4, CTSL, DAAM1, PAICS

## Abstract

The SARS-CoV-2 is the causative agent of the COVID-19 pandemic. The data available about COVID-19 during pregnancy have demonstrated placental infection; however, the mechanisms associated with intrauterine transmission of SARS-CoV-2 is still debated. Intriguingly, while canonical SARS-CoV-2 cell entry mediators are expressed at low levels in placental cells, the receptors for viruses that cause congenital infections such as the cytomegalovirus and Zika virus are highly expressed in these cells. Here we analyzed the transcriptional profile (microarray and single-cell RNA-Seq) of proteins potentially interacting with coronaviruses to identify non- canonical mediators of SARS-CoV-2 infection and replication in the placenta. Despite low levels of the canonical cell entry mediators *ACE2* and *TMPRSS2*, we show that cells of the syncytiotrophoblast, villous cytotrophoblast, and extravillous trophoblast co-express high levels of the potential non-canonical cell-entry mediators *DPP4* and *CTSL*. We also found changes in the expression of *DAAM1* and *PAICS* genes during pregnancy, which are translated into proteins also predicted to interact with coronaviruses proteins. These results provide new insight into the interaction between SARS-CoV-2 and host proteins that may act as non-canonical routes for SARS-CoV-2 infection and replication in the placenta cells.

## Introduction

The severe acute respiratory syndrome coronavirus 2 (SARS-CoV-2) is the causative agent of the coronavirus disease 2019 (COVID-19) (Wu et al., 2020). It was first notified at the end of 2019, in Wuhan, China, and became a worldwide pandemic (Dong E. et al., 2020). At the beginning of September 2021, COVID-19 infected over 227 million people and is the cause of approximately 4.674.673 deaths worldwide (https://coronavirus.jhu.edu/).

Older age, laboratory abnormalities, and several comorbidities are associated with the more severe cases of COVID-19 (Williamson et al., 2020). For specific groups of COVID-19 patients, for example, pregnant women, the potential impacts of SARS-CoV-2 infection remain mostly unknown, and data are limited. However, considering previous works reporting coronaviruses infections (Schwartz and Graham, 2020), pregnant women are at higher risk of SARS-CoV-2 infection due to physiological changes in the immune, cardiorespiratory, and metabolic systems (Qadri and Mariona, 2020).

Although only a small number of maternal virus infections are transmitted to the fetus, some may cause life-threatening diseases (Pereira, 2018). These viruses use cellular host entry mediators expressed by placenta cells, as described for the cytomegalovirus and the Zika virus (Pierson and Diamond, 2018; Hashimoto et al., 2020), to infect these cells. The Zika virus (ZIKV) outbreak, associated with fetal brain damage, emphasizes the necessity of further characterization and understanding of placental infection or intrauterine (vertical) transmission of SARS-CoV-2, as well as the possible adverse fetal outcomes. The few studies on the subject have provided contradictory findings, with some reports suggesting no evidence of placental infection or vertical transmission of SARS-CoV-2 (Celik et al., 2020; Chen et al., 2020; Yang and Liu, 2020). Conversely, multiple lines of evidence have shown placental SARS-CoV-2 infection in pregnant women diagnosed with moderate to severe COVID-19 (Schwartz and Morotti, 2020; Shende et al., 2021; Valdespino-Vázquez et al., 2021). Moreover, neonates born from mothers with COVID-19 presented a positive serological test for SARS-CoV-2 immunoglobulin (Ig)M and IgG (Dong L. et al., 2020; Zeng et al., 2020). While the IgG can be transferred from mother to fetus across the placenta, the detection of IgM in newborns suggests a vertical transmission of the virus, since IgM cannot cross the placental barrier due to its high molecular mass (Kimberlin and Stagno, 2020). Accordingly, SARS-CoV-2 RNA transmission was comprehensively confirmed by pathological and virological investigations (Patanè et al., 2020). Also, it was recently shown SARS-CoV-2 particles in syncytiotrophoblast with generalized inflammation, diffuse perivillous fibrin depositions, and tissue damage in an asymptomatic woman (Schoenmakers et al., 2020). Remarkably, these placental alterations due to the SARS-CoV-2 infection lead to fetal distress and neonatal multi-organ failure. These results highlight the importance of exploring the expression profile of potential host mediators of the SARS-CoV-2 that may create a permissive microenvironment to placental infection and enable vertical transmission of the virus.

In fact, like other viruses, SARS-CoV-2 requires diverse host cellular factors for infection and replication. The angiotensin-converting enzyme 2 (ACE2) is the canonical receptor for the SARS-CoV-2 spike protein receptor-binding domain (RBD) for viral attachment (Letko et al., 2020). This process is followed by S protein priming by cellular transmembrane serine protease 2 (TMPRSS2) that allows the fusion of the virus with host cellular membranes (Letko et al., 2020). Single-cell RNA sequencing (scRNA-Seq) has demonstrated that both *ACE2* and *TMPRSS2* are co-expressed in multiple tissues affected by COVID-19, including airway epithelial cells, cornea, digestive and urogenital systems (Sungnak et al., 2020). Few cells express *ACE2* and *TMPRSS2* in the placenta (Pique-Regi et al., 2020; Sungnak et al., 2020), suggesting that SARS-CoV-2 is unlikely to infect the placenta through the canonical cell entry mediators. Therefore, other host interacting proteins may play a role in the biological cycle of the virus and contribute to the pathogenesis of SARS-CoV-2 in the placenta. In this paper, we demonstrate, through transcriptomic (microarray and scRNA-Seq) analysis and *in silico* predictions of virus-host protein-protein interactions, that cells of the syncytiotrophoblast, villous cytotrophoblast, and extravillous trophoblast express high levels of potential non-canonical cell-entry mediators dipeptidyl peptidase 4 (*DPP4*) and cathepsin L (*CTSL*), despite low-levels of *ACE2* and *TMPRSS2*. We also found changes in the expression of *DAAM1* and *PAICS* genes (translated into proteins predicted to interact with coronaviruses proteins) during pregnancy, which co-express with *DPP4* and *CTSL* in placenta single cells (syncytiotrophoblast, villous cytotrophoblast, and extravillous trophoblast). These results open new avenues of investigation of the human placenta infection by the SARS-CoV-2.

## Results

### Prediction of Non-canonical Routes for SARS-CoV-2 Infection in Human Placenta Cells

**FIGURE 1:**
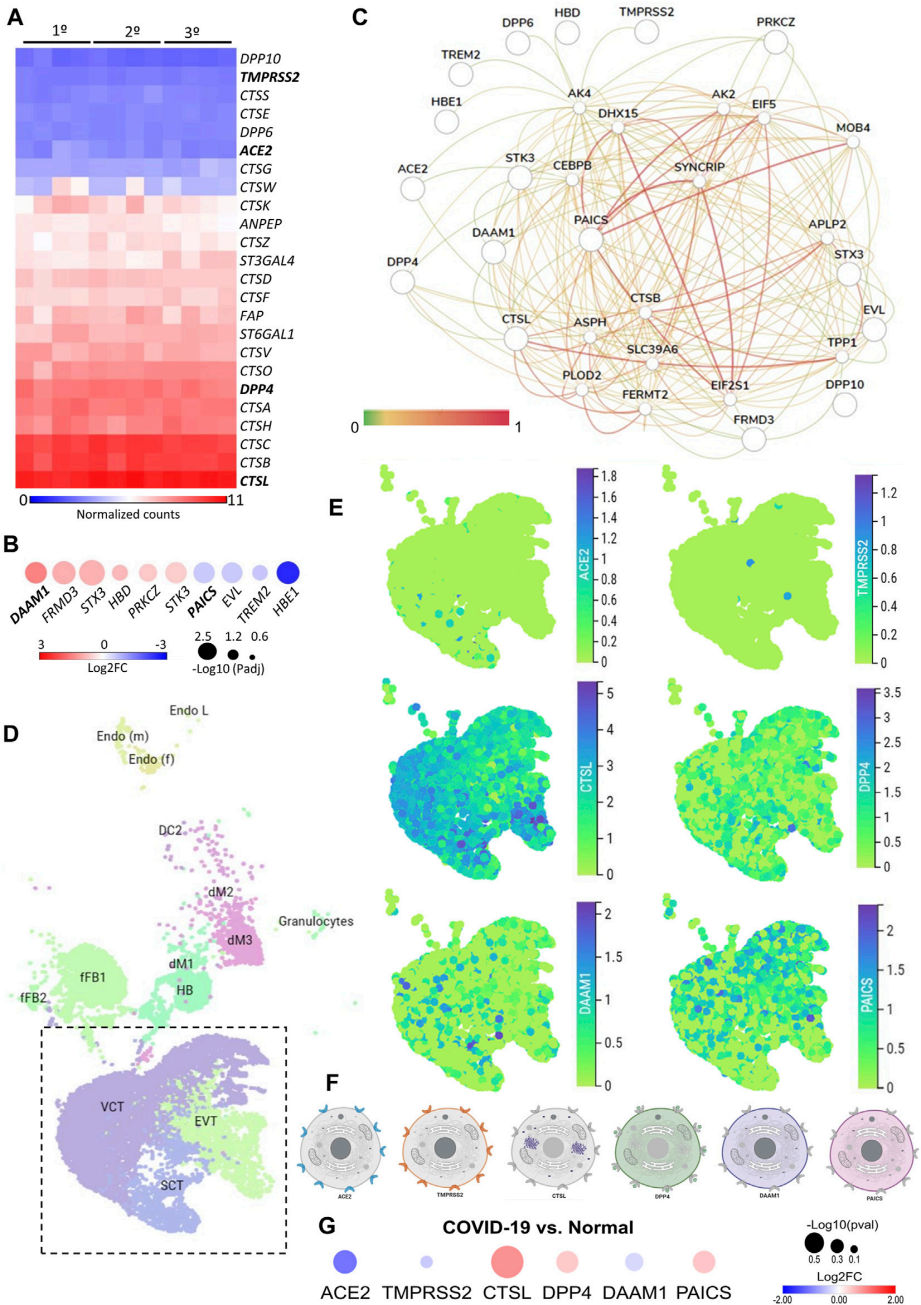
The expression landscape of human placenta proteins potentially interacting with SARS-CoV-2. **(A)**. Heatmap illustrating gene expression (counts RMA normalized) of SARS-CoV-2 interacting proteins from placental samples in the first, second and third trimester of gestation (GSE9984). **(B)**. Heat-scatter plot presenting ten differentially expressed genes in the placenta that encode proteins potentially interacting with SARS-CoV as predicted by the P-HIPSter (http://phipster.org/) database. The color and size of the circles correspond to the log2FC and -log10 transformed FDR adjusted *p*-value, respectively. Fold change represents the gene expression of placenta samples from the third trimester of gestation compared with the first trimester. Bolded genes represent the six candidates selected for the further scRNA-seq evaluations. **(C)**. Tissue-specific gene network of placenta proteins predicted to interact with coronaviruses. The network was generated using the humanbase (https://hb.flatironinstitute.org/) online tool. **(D)**. Dimensionality reduction demonstrates different cell populations identified in five first-trimester human placental samples in a Uniform Manifold Approximation and Projection (UMAP) plot, by using scRNA-Seq data from Vento-Tormo et al., 2018. The images were generated using the COVID-19 Cell Atlas (https://www.covid19cellatlas.org) functionalities. **(E)**. UMAP plots representing the gene expression (log-transformed normalized counts) of *ACE2*, *TMPRSS2*, *CTSL*, *DPP4*, *DAAM1,* and *PAICS* in human placental cells from the syncytiotrophoblast, villous cytotrophoblast, and extravillous trophoblast. The images were generated using COVID-19 Cell Atlas (https://www.covid19cellatlas.org) functionalities. **(F)**. Cell illustrations depicting the protein localization of ACE2, TMPRSS2, CTSL, DPP4, DAAM1, and PAICS in cell compartments, as described in the Human Protein Atlas database (http://www.proteinatlas.org). Illustrations were created using BioRender App (https://biorender.com/). **(G)**. Heat-scatter plot presenting gene expression of *ACE2*, *TMPRSS2*, *CTSL*, *DPP4*, *DAAM1*, and *PAICS* in the placenta from COVID-19 pregnant women compared to healthy pregnant (GSE17995). The color and size of the circles correspond to the log2FC and -log10 transformed *p*-values, respectively. Fold change represents the gene expression of placenta samples from the third trimester of gestation of COVID-19 women compared with matched third trimester uninfected women. RMA: Robust Multichip Average; FC: Fold Change; FDR: false discovery rate; Padj: adjusted *p*-value; SCT, syncytiotrophoblast; VCT, villous cytotrophoblast; EVT, extravillous trophoblast; HB, Hofbauer cells; FB: fibroblasts; dM, decidual macrophages; Endo, endothelial cells; l, lymphatic; m, maternal; f, fetal.

We first investigated the gene expression in placental tissues of classical host-virus interacting proteins described in the literature. The canonical entry receptors *ACE2* and *TMPRSS2* were low expressed throughout gestation. *CTSL*, which is translated into a lysosomal cysteine proteinase that plays a role in intracellular protein catabolism - presented the highest level of expression in the placenta during the first, second, and third trimester. Similarly, *DPP4*, which is translated into an intrinsic membrane glycoprotein, was highly expressed throughout gestation (Figure 1A).

Next, we analyzed the gene expression profile during gestation in placental tissues. We found 25 differentially expressed genes (DEGs) in the second trimester, and 687 DEGs in the third, when they were independently compared to the first trimester (Supplementary Table S1). All DEGs were divided into three clusters by the K-means clustering analysis **(** Supplementary Figure S1A **)**. Cluster 1 includes genes that increase expression during pregnancy, and these genes enriched terms related to blood vessels morphogenesis, complement cascade, extracellular matrix organization, and cellular response to nitrogen compounds. Cluster 2 encompasses genes that increase expression specifically in the third trimesters, which are related to female pregnancy, growth hormone signaling pathway, homeostasis, and steroid biosynthetic process. Cluster 3 includes genes that decrease expression during pregnancy. These genes enriched terms related to cell division, sulfur compounds biosynthetic process, chromosomal segregation, PID MYC active pathway (Supplementary Figure S1B, Supplementary Table S2). The gene expression changes in the course of pregnancy revealed that the second and third trimesters enriched genes that are associated with the placental growth, mainly in the third trimester (Supplementary Figure S1C, Supplementary Table S3). Even with a high discrepancy between the number of DEGs in the second and third trimesters (25 and 687, respectively), we identified similarities in the enriched terms between gene clusters (Supplementary Figure S1D) and between the second and third trimesters of gestation (Supplementary Figure S1E).

We further asked whether the DEGs in the placenta during gestation are translated into proteins that potentially interact with SARS-CoV proteins. Considering that the current cases of SARS-CoV-2 placental infection are mainly related to the third trimester of pregnancy (Patanè et al., 2020; Shanes et al., 2020), we selected the 687 DEGs in the placenta in the third trimester compared to the first trimester and, among them, 474 and 213 were up- and down-regulated, respectively (Supplementary Table S1). Next, we selected the human proteins potentially interacting with SARS-CoV using the P-HIPSter database. We found 32 virus-host interacting proteins of SARS-CoV (Supplementary Table S4), and nine virus-host interacting proteins of the ZIKV (Supplementary Table S5). We also found that, from this list of virus-host interacting proteins, 10 DEGs (*DAAM1*, *FRMD3*, *ST*X3, *HBD*, *PRKCZ*, *CTK3*, *PAICS*, *EVL*, *TREM2*, and *HBE1*) are translated into proteins that interact with SARS-CoV proteins, and one with the ZIKV (Supplementary Figure S1F,G). Among these 10 DEGs, six were up-regulated (*DAAM1*, *FRMD3*, *ST*X3, *HBD*, *PRKCZ*, and *CTK3*) and four (*PAICS*, *EVL*, *TREM2*, and *HBE1*) were down-regulated in the third trimester (Figure 1B **)**. The gene *DAAM1,* which is translated into an intrinsic membrane glycoprotein implicated in cell motility, adhesion, cytokinesis, and cell polarity—showed the highest level of fold change in the third trimester of gestation compared to the first (logFC = 1.43; Figure 1B). The host-virus protein-protein interactions (PPI) predicted for these 10 DEGs presented a Likelihood Ratio (LR) > 100 (Supplementary Table S6), according to P-HIPSTer (Lasso et al., 2019). Noteworthy, *PAICS* transcript, which is translated into an enzyme that catalyzes the sixth and seventh steps of the novo purine biosynthesis, were predicted to interact with both SARS-CoV and ZIKV (Figure 1B, Supplementary Table S7). We observed a significant interaction of placental proteins predicted to interact with SARS-CoV-2 (based on the DEGs or not), and PAICS was also predicted to interact with other proteins in the PPI network with the highest degree (Figure 1C, Supplementary Table S8).

**FIGURE 2:**
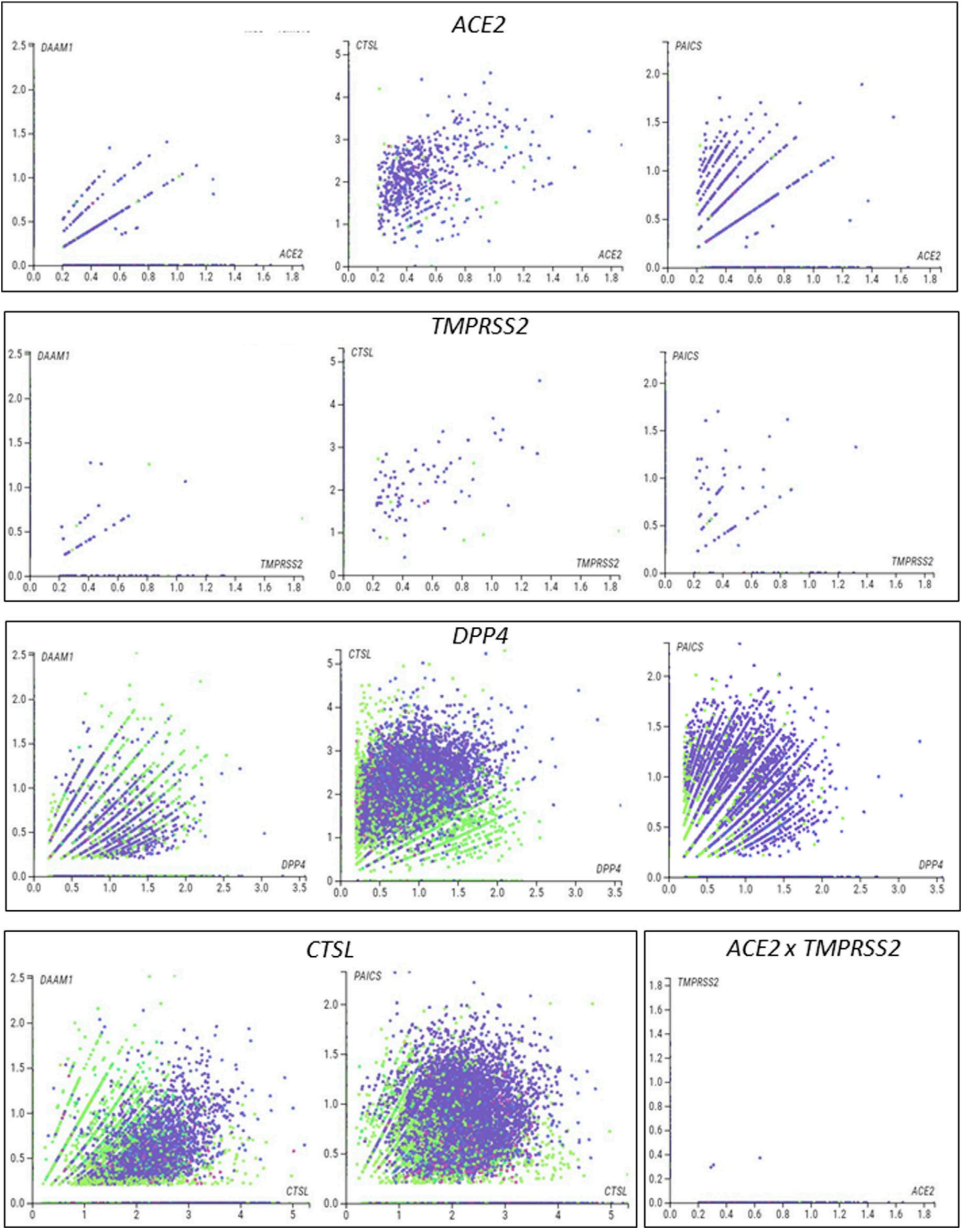
Single-cell analysis of the human placenta indicates potential non-canonical routes of SARS-CoV-2. **(A)**. Co-expression analysis of *DAAM1*, *CTSL*, DPP4, and *PAICS* with the canonical virus entry-associated genes *ACE2* and *TMPRSS2*. **(B)**. Co-expression analysis of *DAAM1* and *PAICS* with the non-canonical virus entry genes *DPP4* and *CTLS*. The images were generated using COVID-19 Cell Atlas (https://www.covid19cellatlas.org) functionalities.

We next used single-cell RNA sequencing to analyze the expression of the 10 DEGs translated into proteins that potentially interact with SARS-CoV in human placental cells (Supplementary Figure S1H,I). We selected the genes *ACE2*, *TMPRSS2*, *CTSL*, *DPP4, PAICS,* and *DAAM1* for further investigations using scRNA-seq transcriptome data from cells of the syncytiotrophoblast (*n* = 1,144), villous cytotrophoblast (*n* = 8,244), and extravillous trophoblast (*n* = 2,170) of non-disease human placental tissues, considering the potential relevance of these genes for SARS-CoV infection and replication in the organ (Figure 1D). We noticed that the expression of the classical SARS-CoV-2 entry receptor genes (*ACE2* and *TMPRSS2*) was minimally expressed in these cells. In contrast, potential non-canonical cell entry mediator genes *DPP4* and *CTSL*, as well as the genes for the predicted virus-host interaction proteins *DAAM1,* and *PAICS* were expressed at higher levels (Figure 1E, Supplementary Figure S2A). We also looked for the co-expression of *ACE2* and *TMPRSS2* with *DPP4*, *CTSL*, *DAAM1,* and *PAICS* by using scRNA-Seq in these same cells (Figure 2). This analysis revealed that *DPP4, CTSL, DAAM1*, and *PAICS* are co-expressed at high levels, while these genes are co-express at low levels with *ACE2* and *TMPRSS2* (Figure 2). Remarkably, we found only three cells co-expressing *ACE2* and *TMPRSS2*. For this reason, we consider that *DPP4, CTSL, DAAM1*, and *PAICS* may represent candidates of an alternative route for SARS-CoV-2 infection in human placentas. We used The *Human Protein Atlas* to predict the subcellular location of proteins encoded by our candidates. DPP4 is predicted as intracellular, membrane, and secreted protein; CTSL is a protein located in the Golgi apparatus and additionally in vesicles; PAICS is a protein found in the cytosol while DAAM1 is in the plasma membrane and cytosol (Figure 1F). A recent published study investigated the gene expression alteration in the term placentas from COVID-19 pregnant women compared to uninfected (Lu-Culligan et al., 2021). We have accessed the DEGs identified by the authors, and we found that our candidate genes are not differentially expressed (Figure 1G). Moreover, *DAAM1* and *PAICS* are expressed in the opposite direction to the one found during gestational ages (Figure 1B). Finally, we analyzed the gene expression profile of *ACE2*, *TMPRSS2*, *CTSL*, *DPP4*, *DAAM1,* and *PAICS* genes on publicly available scRNA-Seq datasets of lung, liver, and thymus fetal tissues. *CTSL*, *DPP4,* and *DAAM1* were found as highly expressed in fetal cells compared to *ACE2* and *TMPRSS2* in all tissues analyzed (Supplementary Figure S2).

## Discussion

Recent literature has shown placenta cells infected with SARS-CoV-2 in pregnant women diagnosed with moderate to severe COVID-19 (Patanè et al., 2020; Penfield et al., 2020), with findings supporting the vertical transmission of SARS-CoV-2 in early and late pregnancy (Hosier et al., 2020; Patanè et al., 2020; Schwartz and Morotti, 2020; Shende et al., 2021; Valdespino-Vázquez et al., 2021). However, few placenta cells express *ACE2* and *TMPRSS2* (Pique-Regi et al., 2020; Sungnak et al., 2020) required for virus entry, raising the question of whether potential non-canonical molecular mechanisms may be involved in the biological cycle of the virus in these cells. Here, we demonstrated by transcriptomic analyses (microarray and scRNA-Seq) and *in silico* predictions of virus-host protein-protein interactions that, despite low-levels of *ACE2* and *TMPRSS2*, villous trophoblast cells express high levels of the potential non-canonical cell-entry mediators *DPP4* and *CTSL*. We also found changes in the expression of *DAAM1* and *PAICS* genes that code for proteins predicted to interact with SARS-CoV proteins during pregnancy. These results provide evidence of potential host-virus PPI that may lead to SARS-CoV-2 infection and replication in the human placenta.

Firstly, we characterized the expression of SARS-CoV-2/SARS-CoV entry-associated receptors and proteases genes, which revealed that *DPP4* and *CTSL* are highly expressed in villous trophoblast cells during pregnancy. Similar to our findings, *DPP4* and *CTSL* expression was already identified in first and second trimester human placenta single cells (Ashary et al., 2020). DPP4 has a high affinity to the SARS-CoV-2 spike receptor-binding domain, (Li et al., 2020), and critical DPP4 residues share the SARS-CoV-2-S/DPP4 binding as found for MERS-CoV-S/DPP4 (Hoffmann et al., 2020). Although it was demonstrated that DPP4 is not a SARS-CoV-2 entry receptors in BHK-21 cells (Hoffmann et al., 2020), more studies are necessary to address the role of *DPP4* in placental cells. In a pioneer study based on SARS-CoV cell entry mechanisms, (Simmons et al., 2005) described a three-step method for host-virus membrane fusion, involving receptor-binding and induced conformational changes in S glycoprotein with subsequent CTSL proteolysis and activation of membrane fusion within endosomes. During the COVID-19 pandemic, it has been further demonstrated that SARS-CoV-2 can use CTSB and CTSL as well as TMPRSS2 for priming host cells (Hoffmann et al., 2020; Ou et al., 2020). These data provide evidence that CTSL is a potentially promising treatment for COVID-19 by blocking coronavirus host cell entry and intracellular replication (Liu et al., 2020). Secondly, we analyzed whether placental development and growth are associated with transcriptional changes in proteins that potentially interact with SARS-CoV. Among the transcripts translated into proteins predicted as interacting with SARS-CoV, *DAAM1* increased with placental development and growth. DAAM1 was previously identified as a regulator of bacteria phagocytosis by regulating filopodia formation and phagocytic uptake in primary human macrophages (Hoffmann et al., 2014). We predicted that DAAM1 potentially interacts with the viral protein encoded from open reading frame 8 (ORF8) and the hypothetical protein sars7a of SARS-CoV. The SARS-CoV ORF8 shares the lowest homology with all SARS-CoV-2 proteins; however, it was previously demonstrated that SARS-CoV-2 ORF8 expression was able to selectively target MHC-I for lysosomal degradation by an autophagy-dependent mechanism in different cell types (Zhang et al., 2020). We found that the *PAICS* transcript, which is also translated into a protein predicted as interacting with the Nsp3 of SARS-CoV, decreased its levels with placental development and growth. PAICS is an enzyme of the *de novo* purine biosynthesis pathway, which was previously predicted as having a putative interaction with the human influenza A virus (IAV) nucleoprotein and was up-regulated during IAV infection (Generous et al., 2014). The putative interaction of Nsp3 with PAICS is relevant. Nsp3 is one of the 16 non-structural proteins in the replicase ORF1ab gene of SARS-CoV and SARS-CoV-2 (Enjuanes, 2005; Wu et al., 2020), and binds to virus RNA and proteins, including the nucleocapsid protein (Lei et al., 2018). Considering the low levels of *ACE2* and *TMPRSS2* that we found in villous trophoblast cells, our results provide evidence of alternative cell-entry mediators in the placenta. Moreover, the description of the potential interaction between host and SARS-CoV-2 may provide insights into the mechanisms of placental infection in women with COVID-19.

Cellular and molecular composition play a role in placenta infection and intrauterine transmission of viruses (Koi et al., 2001; Barbeito-Andrés et al., 2020). Thus, we finally used single-cell analysis of the syncytiotrophoblast, villous cytotrophoblast, and extravillous trophoblasts—the fetal component of the placenta—that confirmed the low levels of expression of the canonical entry receptor *ACE2* and *TMPRSS2* genes (Pique-Regi et al., 2020; Sungnak et al., 2020). Conversely, we found that these cells express high-levels of *DPP4* and *CTSL*, two potential mediators of SARS-Cov-2 host cell entry (Hoffmann et al., 2020; Li et al., 2020) that may contribute to the SARS-CoV-2 infection (Hosier et al., 2020; Patanè et al., 2020; Penfield et al., 2020). It is essential to highlight that our scRNA-Seq analysis showed that the non-canonical *DPP4* and *CTSL* entry genes (Hoffmann et al., 2020; Li et al., 2020) are highly co-expressed in syncytiotrophoblast, villous cytotrophoblast, and extravillous trophoblasts cells. *DPP4* and *CTSL* expression landscape in different tissues were extensively investigated in scRNA-seq study (Singh et al., 2020). However, there was no evidence of *DAAM1* and *PAICS* role. We found that the transcripts *DAAM1* and *PAICS*, which are translated into proteins predicted as potentially interacting with SARS-CoV-2, were also highly co-expressed with *DPP4* and *CTSL.* These results demonstrate that, although *ACE2* and *TMPRSS2* are poorly expressed in the placenta, other mediators that potentially interact with the virus are highly co-expressed in villous trophoblast cells and, therefore, may represent a valuable alternative route for infection and viral replication.

Although our *in-silico* analyses are a starting point, they have limitations. We reanalyzed published placenta datasets with a limited number of samples (microarray = 12 individuals, and scRNA-seq = 5 individuals) and, consequently, validations in a large cohort of samples must be performed. We understand that under sampling in transcriptomic data are a limitation and statistical analysis as imbalanced learn and ablation tests would benefit our methodology, however, our main results are supported by the relevant literature. Moreover, the predicted interactions presented here should also be experimentally validated in infected cells or placenta to circumvent these limitations. Moreover, the analyses of single-cell data should be conducted for the entire period of gestation, considering that we only evaluated single-cell data from the first trimester of pregnancy. The detection of SARS-CoV-2 in the placenta needs to be performed in a large cohort of pregnant women with COVID-19, including asymptomatics. Considering the vertical transmission of SARS-CoV-2, the follow-up of newborns from mothers with COVID-19 during pregnancy should be necessary since, if it occurs even in the non-asymptomatic, the long-term consequences are mostly unknown.

In conclusion, despite low levels of *ACE2* and *TMPRSS2*, our analyses demonstrate that villous trophoblast cells express high levels of potential non-canonical cell-entry mediators *DDP4* and *CTSL.* We also found changes in the expression of the *DAAM1* and *PAICS* genes coding for proteins predicted to interact with SARS-CoV proteins during pregnancy. These results provide new insight into the interaction between SARS-CoV-2 and the host proteins and indicate that coronaviruses may use multiple mediators for virus infection and replication.

## Materials and Methods

### Differential Expression Analysis of the Placenta During Pregnancy Trimesters

We reanalyzed microarray data from villous trophoblast tissues of first (45–59 days, *n* = 4), second trimester (109–115 days, *n* = 4), and third trimesters (*n* = 4) of uncomplicated pregnancies, available in Gene Expression Omnibus (GEO), under the accession number GSE9984 (Mikheev et al., 2008). Next, we identified the DEGs during the pregnancy, by comparing the second trimester and third trimester with the first trimester using the GEO2R tool (https://www.ncbi.nlm.nih.gov/geo/geo2r ). We applied the default settings of GEO2R to perform transcriptome analysis on original submitter-supplied processed data tables (RMA normalized signal log2) using the GEOquery and limma R packages from the Bioconductor project. Genes with Log2 of Fold Change ≥ |0.5| and False Discovery Rate (FDR) < 0.05 were considered as DEGs. We further grouped the gene expression profiles during pregnancy by using the K-means clustering analysis based on One Minus Pearson Correlation (Robust Multi-array Average, RMA, normalization log2, K-means = 3).

### Enrichment Analysis

We used the Metascape tool (https://metascape.org) (Zhou et al., 2019) to perform functional enrichment analysis of the genes lists generated in the clustering and differential expression analyses.

### Placenta Proteins That Potentially Interact With Coronaviruses and Zika Virus

SARS-CoV-2 cell entry mediators were selected for first screening using the human proteins already described in the COVID-19 Cell Atlas (https://www.covid19cellatlas.org/) and the literature (Hoffmann et al., 2020; Li et al., 2020; Sungnak et al., 2020; Zhou et al., 2020)*.* Next, we used gene expression data to generated a list of PPIs that potentially interact with human coronaviruses and the ZIKV from the Pathogen–Host Interactome Prediction using Structure Similarity (P-HIPSTer, http://phipster.org/) database (Hosier et al., 2020) (Supplementary Tables S4,S5, respectively). SARS-CoV was included in the analysis considering the evolutionary relationship between the novel SARS-CoV-2 (Zhou et al., 2020), and ZIKV considering its impact on placental infection and fetal microcephaly (Pierson and Diamond, 2018; Barbeito-Andrés et al., 2020).

### Tissue-specific Gene Networks of Potential Virus-Host Placenta Interactome

The humanbase webtool (https://hb.flatironinstitute.org) (Greene et al., 2015) was used to generate the tissue-specific gene network of placental genes coding for proteins that potentially interact with SARS-CoV-2 based on P-HIPSTER or that we identified as either associated with COVID-19 or that we hypothesized may be associated with the disease, based on the literature (Supplementary Table S8). We considered co-expression, interaction, transcriptional factor binding, Gene Set Enrichment Analysis (GSEA) perturbations as active interaction sources, applying minimum interaction confidence of 0.07, and a maximum number of 15 genes.

### Single-Cell Transcriptome Analysis of Human Placentas and Fetal Tissues

We used the functionalities of the COVID-19 Cell Atlas (https://www.covid19cellatlas.org) to explore single-cell RNA sequencing (scRNA-Seq) data from five samples from first-trimester placentas, previously described by Vento-Tormo et al. (2018). The processed scRNA-seq data (log-transformed normalized counts) of placental genes potentially interacting with SARS-CoVs was visualized in placental cell types using the “interactive viewer” developed by cellxgene v0.15.0 (https://cellxgene.cziscience.com) by the Chan Zuckerberg Initiative available at https://cellxgene.cziscience.com/d/fetal_maternal_interface_10x-22.cxg/. We also characterized the gene expression profile of canonical and non-canonical placental mediators of viruses interaction per cell using cellxgene v0.15.0 bar plots. The placental single-cell transcriptome is available for re-analysis at Human Cell Atlas Data Portal (https://data.humancellatlas.org) or E-MTAB-6701 (EMBL-EBI, ArrayExpress; https://www.ebi.ac.uk/arrayexpress). We applied this same strategy to investigate the gene expression of our candidates using scRNA-seq in human fetal tissues: lung, liver, and thymus (ArrayExpress; E-MTAB-8221, E-MTAB-7407, and E-MTAB-8581, respectively. Last accessed June 2020.

### Placenta RNA Sequencing From Pregnant Women With COVID-19

We downloaded the list of differentially expressed genes from 3rd trimester placental villi of pregnant women with COVID-19 (*n* = 5) compared with uninfected control individuals matched for maternal age, gestational age, maternal comorbidities, and mode of delivery (*n* = 3), publicly available at GSE17995 (Lu-Culligan et al., 2021). The authors conducted the differential expression analysis using DESeq2 v1.24.0 with default parameters, and we downloaded the CSV file from GEO.

### Data Representation and Analysis

Morpheus (https://software.broadinstitute.org/morpheus/) was used to generate the heatmaps and to perform the clustering analyses. The Venn diagram was constructed using the Van de Peer Lab software (http://bioinformatics.psb.ugent.be/webtools/Venn/). Biorender was used to design cell images (https://app.biorender.com/).

## Data Availability

The datasets presented in this study can be found in online repositories. The names of the repository/repositories and accession numbers can be found in the article/Supplementary Material.
